# Volumineux corps étranger intra-rectal: à propos d'un cas

**DOI:** 10.11604/pamj.2014.18.273.4226

**Published:** 2014-08-04

**Authors:** Khalid Mazine, Abdesslam Bouassria, Hicham Elbouhaddouti, Ouadii Mouaqit, Elbachir Benjelloun, Abdelmalek Ousadden, Khalid Mazaz, Khalid Ait Taleb

**Affiliations:** 1 Service de Chirurgie Viscérale, CHU Hassan II – Fès, Université Sidi Mohamed Ben Abdellah, Faculté de Médecine et de Pharmacie de Fès, Fès, Maroc

**Keywords:** Corps étranger, rectum, voie anale, foreign body, rectum, annaly

## Abstract

L'insertion d'objets dans le rectum est peu courante dans les pays au contexte socio-culturel tels que le Maroc. Elle se caractérise par la gravité des complications éventuelles et les différentes possibilités thérapeutiques. Nous rapportons le cas d'un patient, ayant eu une incarcération d'un énorme objet introduit volontairement par voie anale, cet objet étant soigneusement façonné par le patient. Il a bénéficié d'une extraction manuelle. Cette dernière permet, quand elle est possible, d’éviter la chirurgie qui s'impose en cas d’échec ou de complications.

## Introduction

L'introduction de corps étrangers (CE) par l'anus est un phénomène bien décrit qui reste, au Maroc, une curiosité et un tabou. On ne peut donc pas estimer la fréquence de cette pathologie. Des objets peuvent être introduits dans le rectum à des fins thérapeutiques, sexuelles (érotisme anal ou agressions sexuelles), par trouble du comportement, pour dissimuler l'objet (drogues, etc) ou plus rarement, lors de circonstances accidentelles. Ces CE sont de nature très diverse et insolite (Bouteille, déodorant, magazine, etc).

## Patient et observation

**Figure 1:**
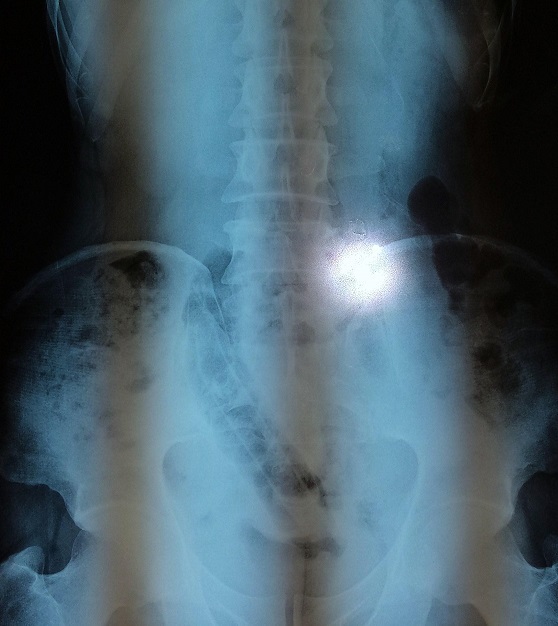
Abdomen sans préparation montrant le corps étranger au niveau du pelvis

**Figure 2:**
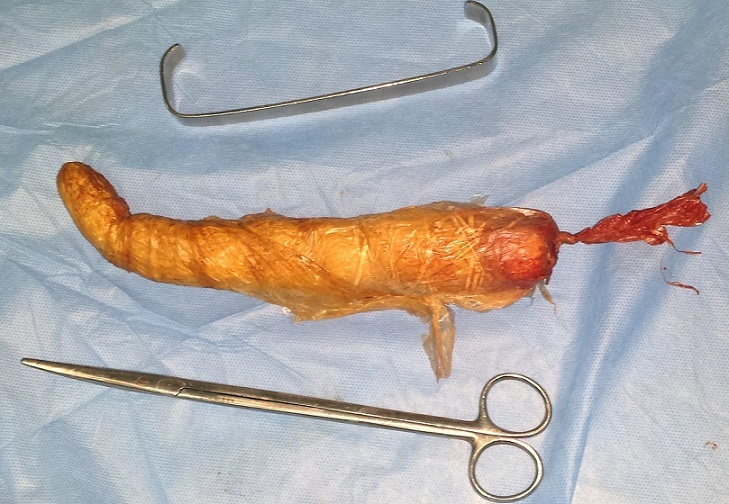
Corps étranger après son extraction

**Figure 3:**
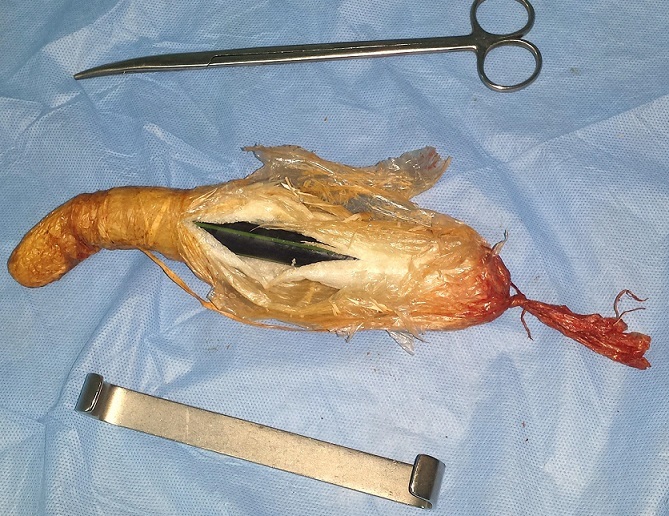
Corps étranger après son extraction

Un patient de 49 ans sans antécédents notables, s'est présenté aux urgences pour une sub-occlusion en rapport avec un CE incarcéré en intra-rectal depuis son introduction cinq jours auparavant en vue de traiter une crise hémorroïdaire. L'examen trouvait un patient en bon état général, stable sur le plan hémodynamique, et apyrétique. L'examen abdominal était sans particularités, avec un abdomen souple et non distendu. Le toucher rectal percevait au bout du doigt l'extrémité distale de l'objet qui venait buter contre l'excavation sacrée. La radiographie de l'abdomen sans préparation permettait de visualiser le CE, volumineux et se projetant au niveau du pelvis (Figure 1). L'extraction par voie basse à l'aide d'une pince a été faite sous sédation, au bloc opératoire, en position de taille périnéale. Il s'agissait d'un objet volumineux et oblong, mesurant près de 15 cm, ayant été fabriqué par le patient à partir d'un tuyau d'arrosage soigneusement recouvert de plusieurs couches de plastique, et introduit volontairement (Figure 2, Figure 3). Le patient était gardé en observation après l'extraction. L’évolution était sans particularités, et la reprise d'un transit effectif en gaz et en selles s'est faite le lendemain de l'extraction. Le patient était déclaré sortant J + 2.

## Discussion

Le rapport le plus ancien sur la prise en charge d'un corps étranger intra-rectal remonte au XVIème siècle [1]. On distingue l'incarcération de corps étrangers ingérés par voie buccale et les corps étrangers introduits par voie rectale pour diverses raisons. La cause la plus fréquente d'insertion de corps étranger est liée aux pratiques sexuelles, la plupart du temps solitaires. Les autres étiologies sont l'auto-thérapeutique (d'une constipation, d'hémorroïdes, ou de prurit anal), l'origine traumatique, les agressions, et l'origine psychiatrique [2]. Notre patient rapporte l'introduction du corps étranger dans un but d'auto-thérapeutique, même si l'hypothèse de pratique sexuelle solitaire ne peut être écartée. La présence de corps étranger intra-rectaux est très peu courante dans les pays en développement, et plus fréquente dans les pays industrialisés [3]. Les patients se présentent souvent aux urgences plusieurs heures ou plusieurs jours après l'insertion du corps étranger, avec un délai moyen de 1,9 jours [1]. Notre patient s'est présenté 5 jours après l'introduction du corps étranger. Les principaux motifs de consultation sont la rectorragie et la douleur abdominale aigüe ou persistante associée à un syndrome occlusif ou sub-occlusif. Les ténesmes ou les inconforts ano-rectaux sont souvent citées [4]. Un toucher rectal (mieux réalisé sous sédation), vérifie l'intégrité anorectale et peut retrouver le CE. Combiné à la palpation abdominale, il permet parfois d'estimer sa position [5]. Si l'objet est radio-opaque, le diagnostic est confirme à l'ASP qui visualise sa forme, sa taille et sa position. L'ASP peut aussi objectiver un pneumopéritoine, signe d'une perforation digestive, imposant la laparotomie en urgence. Les corps étrangers peuvent en effet être à l'origine d’érosions intestinales ou vasculaires, d'abcès, d'obstruction, et d'hémorragie [6]. Si le diagnostic est établi avant le stade de complication, l'extraction s'imposerait sauf que cette dernière pose aussi une problématique. Elle doit être réalisée par voie basse dans la mesure du possible. Une anesthésie locorégionale ou même générale au bloc opératoire s'impose pour un relâchement des sphincters anaux [7]. Des succès d'extraction par voie basse ont été rapportés mais concernent surtout les CE de petite taille [7]. Chez notre patient, malgré la taille considérable de l'objet, l'extraction par voie basse était réussie. Certains facteurs comme la taille, la forme et la migration des corps étranger peuvent rendre difficile la recherche et l'extraction par voie basse. En cas d’échec, une laparotomie s'avère nécessaire [8, 9] pour repousser l'objet vers l'ampoule rectale sans ouvrir le colon. Néanmoins, pour les corps étrangers de grande taille, une colostomie peut s'avérer nécessaire. [10]. La mise en place d'une stomie d'amont dépend du traumatisme périnéal, de la chronicité de la situation, et de l’état de la paroi colorectale appréciée en per opératoire [11].

## Conclusion

Les corps étrangers colorectaux introduits par voie anale sont des évènements qui restent peu fréquents dans notre contexte, mais les praticiens exerçant dans nos contrées peuvent y être confrontés. Cette observation en a illustré un cas montrant la possibilité de l'extraction par voie basse malgré la taille considérable du corps étranger. Si le diagnostic est relativement facile après un interrogatoire soigneux et un examen clinique rigoureux, une prise en charge rapide permet d’éviter de graves complications.
